# The Profile of Moods States and Athletic Performance: A Meta-Analysis of Published Studies

**DOI:** 10.3390/ejihpe11010005

**Published:** 2021-01-13

**Authors:** Marc Lochbaum, Thaís Zanatta, Deylon Kirschling, Emily May

**Affiliations:** 1 Department of Kinesiology and Sport Management, Texas Tech University, Lubbock, TX 79409, USA; 2 Education Academy, Vytautas Magnus University, 44248 Kaunas, Lithuania; 3 Psychological Sciences, University of California, Merced, CA 95343, USA; tbenoit2@ucmerced.edu; 4 College of Rehabilitative Sciences, University of St. Augustine Health Sciences, Austin, TX 78739, USA; d.kirschling@usa.edu; 5 School of Health Professions, University of Texas Medical Branch, Galveston, TX 77555, USA; ecmay@utmb.edu

**Keywords:** mental health model, iceberg profile, vigor, depression, total mood disturbance, quantitative review

## Abstract

Researchers have extensively examined and reviewed the relationship of the profile of mood states (POMS) with sport performance since the 1970s. Two decades have passed since the last POMS quantitative review. Our overall objective was to quantify the POMS research with competitive athletes with a prospective measured POMS and a sport performance outcome in the published literature. Additionally, we tested potential moderators of the mental health model (i.e., sport duration, type, and skill) with meta-analytic techniques while considering potential risk bias across study sources. Based on a systematic review, the articles were found using EBSCO and comparing these articles with extensive past POMS in sport and exercise bibliographies. Search terms included profile of mood states (POMS) or iceberg profile or the mental health model with sport and performance or sports performance. For selection, articles must have reported data on competitive athletes, an athletic performance outcome, and a valid form of the POMS measured prospectively. After screening more than 600 articles for inclusion, 25 articles provided sufficient data for effect size calculations. The included articles spanned from 1975 to 2011, with 1497 unique participants. Hedges’ g values were generally small for the six POMS scales: tension (−0.21), depression (−0.43), anger (−0.08), vigor (0.38), fatigue (−0.13), and confusion (−0.41). However, the total mood disturbance (TMD) score effect size was medium in magnitude at −0.53. When corrected for potential publication bias, the effect size values increased in magnitude for tension (−0.47), depression (−0.64), vigor (0.44), fatigue (−0.34), and TMD (−0.84). Moderator analyses for Terry’s (1995) propositions and for risk of bias across studies, statistically, resulted in few differences based on conventional statistical significance (*p* < 0.05). Measured before performance, most of the POMS scales and TMD are reliable predictors of sport performance in competitive athletes across a wide variety of sports and athletic performance outcomes. Morgan’s (1980, 1985) mental health model or iceberg profile minus anger is still a viable method for understanding and improving athletic performances.

## 1. Introduction

Given that sport has performance statistics and winners and losers, an understanding of how to affect performance and thus the outcome of sporting events is valued and researched across all sport science areas. In sport psychology research, understanding athletic performance predictors is a main goal and there is much literature on the subject. For instance, researchers have quantified many sport psychology interventions and constructs (see Lochbaum [1]) relating (to) athletic performance such as goal setting [2], achievement goals [3], mental practice [4], and team cohesion [5]. As with the above-mentioned sport psychology and performance topics, mood states as measured by the profile of mood states (POMS) questionnaire [6] is a much-researched topic within sport psychology under Morgan’s [7,8] mental health model or often termed iceberg profile. Indeed, two meta-analyses have been previously published [9,10] as well as a number of systematic reviews [11,12,13,14].

Given the importance of predicting performance, the longevity of Morgan’s mental health model with the POMS in sport psychology research, and the passing of at least 20 years since the last reviews [10,11,12,13], we extensively searched the peer-reviewed published POMS and sport performance research. This review only included POMS studies using McNair and colleagues’ [6] measure. We then meta-analyzed data when available, examined potential sources of bias, and used mixed-effects analysis techniques to test a number of hypotheses with the hopes of solidifying and furthering POMS research in the competitive sport environment regarding predicting performance.

### 1.1. Morgan’s Mental Health Model

Morgan and his colleagues appeared to begin using the POMS shortly after its publication [6] to predict US Olympic wrestlers’ trial success [15,16] and rowing [17]. Within the next decade, Morgan [7], in his Psychology Today popular press article, termed the unique differences found between successful and unsuccessful sport performers based on the POMS subscales, with successful performers lower in tension, depression, anger, fatigue, confusion, and higher in vigor compared to less successful performers, the iceberg profile. Researchers enthusiastically took to the POMS in competitive sport contexts to explain sport performances in both individual sports such as distance running [18], speed skating [19] and team sports such as football [20] and netball [21]. It was in this time period, in an edited text titled Limits of Human Performance, that Morgan [8] presented his mental health model, stating “positive mental health enhances the likelihood of success in sport, whereas psychopathology is associated with a greater incidence of failure” [8] (p. 79).

By the 1990s, enough research via academic conference presentations and published articles had accumulated for both qualitative [11,12] and quantitative [9] reviews. In the next decade, two qualitative [13,14] and one quantitative [10] reviews kicked off the new decade. A number of inconsistencies pervaded the reviews. For instance, since the first review [11,12], the notion of the POMS accurately or significantly discriminating among levels of successful athletes or compared to non-athletes has been viewed as unsubstantiated as well as “entirely unreasonable” [12] (p. 310).

Renger [11] pointed his criticism to Morgan’s [7] overly simplified popular press article as well as using the POMS as a personality measure. Terry [12] stressed, in a more positive light, that research to best understand the mood and performance relationship both between and within athletes is needed. He highlighted a number of areas, such as early problem identification, load monitoring, and the like. Further, in 1995, Rowley and colleagues [9] (most notably his mentor Daniel Landers) published the first meta-analysis on the POMS. Though casting a less than supportive conclusion “...the utility of the POMS in predicting athletic success is questionable” [9] (p. 185), the overall effect size, representing the POMS total mood score, though small (ES = 0.15), was statistically different from zero, indicating that more successful athletes’ mood profiles conformed to Morgan’s iceberg profile.

By the time of the next round of POMS reviews, research with the POMS flourished. For instance, LeUnes and Burger [13] identified 258 published articles using the POMS in sport and exercise contexts based on LeUnes’ updated POMS bibliographies [22,23,24]. LeUnes and Burger [13] concluded with improved research techniques and POMS measurement (e.g., validated measure for youth) that “mood profiling will be important components of POMS research and application into the millennium” [13] (p. 14). In contrast, Prapavessis [14], in his review, pushed for Hanin’s Individual Zone of Optimal Function model (see Ruiz, Raglin, and Hanin [25] for a historical review of Hanin’s work) over Morgan’s mental health model to best understand sport performance.

Beedie and colleagues’ [10] meta-analysis provided more definitive answers by separating level of achievement studies and performance outcome studies. They coded and attempted to analyze their data based on a number of Terry’s [12] mood and performance propositions such as sport type and duration, operational definition of performance success, timing of mood assessment relating (to) performance, and POMS response set. Additionally, unlike Rowley and colleagues’ [9] work, Beedie et al. [10] reported data for all POMS subscales when available. For the achievement studies, when averaged together appropriately (all coded to positive mental health), the overall effect size (0.10) was nearly identical to that of Rowley’s. Separated, the vigor effect size (0.20) was the largest value supporting Morgan’s iceberg profile.

The change in tone concerning support of Morgan’s mental health model or iceberg profile stemmed from the mood and performance results. Across 17 samples, the effect size values in order of magnitude were 0.47 (vigor), −0.40 (confusion), −0.34 (depression), −0.27 (anger), −0.25 (tension), and −0.13 (fatigue). Citing small samples across their potential moderator variables based on Terry’s [12] propositions, Beedie and colleagues [10] reported effect size representing total mood values without statistical testing for differences. Those values, regardless of the proposition category, ranged from 0.27 to 0.39, thus all still being categorized as small [26]. Though subscale effect size values are found in their review (see Table 2, p. 62), the authors did not expound upon them. Even with limited samples and statistical tests, Beedie and colleagues [10] concluded in their abstract based concerning the POMS predicting performance outcomes, “the POMS has utility” [10] (p. 49) though, in their discussion, they qualified this utility as “moderate at best” [10] (p. 63). Certainly, one could argue the subscale effect size values approaching moderate in magnitude are of value concerning bettering an athlete’s performance.

### 1.2. Objectives

In summary, improving athletic success in competitive sport is valued. Thus, based on our examination of the POMS reviews and the potential for more advanced statistical testing, our overall objective was to update the research knowledge since Beedie and colleagues’ [10] meta-analysis concerning Morgan’s mental health model [7,8] as measured by the POMS related only to predicting athletic success. To achieve our overall objective, we reviewed all found published studies that specially assessed the POMS before athletic performance in competitive settings with competitive athletes. By doing so, we tested whether the mental health model or iceberg profile is still characteristic of successful performances. Then we investigated a number of Terry’s [12] proposed moderators that received mixed support in the Beedie and colleagues’ [10] meta-analysis. We placed Terry’s proposition concerning performance references (i.e., objective or self-referenced) as a potential risk of across-study bias moderators.

### 1.3. Research Questions

Specifically, we tested the following research questions. In Morgan’s [7,8] mental health model, especially higher levels of vigor and lower levels of tension, depression, anger, fatigue, and confusion, characterize successful athletic performances and are invariant to potential across study risks of bias. Based on Terry’s [12] proposed moderators, we tested whether the POMS subscales explain (i.e., larger in effect size values) successful athletic performance more in (a) short-duration sports—defined as less than 10 min, when compared to longer-duration sports; (b) closed-skill sports—defined as mostly self-paced, little to the interaction between or among competitors, and few external performance influences when compared to open-skill sports, which are defined as sports with higher unpredictable competitor interactions or potential external influences; and (c) individual sports—defined as sports requiring no teammate cooperation, compared to team sports, which are defined as sports requiring teammate cooperation, whether open- or closed-skill sports. Across all three moderators, the notion is that mood, as measured by the POMS, before the sporting event should be more accurate of mood within the event because the event is shorter in time or has fewer outside influences or no teammates to impact competition mood and thus potentially performance.

## 2. Materials and Methods

This meta-analysis reported on each PRISMA statement item [27]. Thus, our reporting is transparent with the goal of being perceived as comprehensive.

### 2.1. Protocol and Registration

Researchers have tested Morgan’s mental health model or iceberg profile since the 1970s, and Terry’s proposed moderators are of written record. Thus, we did not register our protocol in a database. We specified our search strategy, inclusion criteria, data extraction, and data analyses in advance of writing our manuscript. All details of our work, if in question, are available from the lead author.

### 2.2. Eligibility Criteria

Articles retained for extensive examination met the following inclusion criteria: papers with (a) any methodological design such as mean group, between or within differences or correlates with performance; (b) a publication date after that of the POMS up to 1 January 2020; (c) original data published in peer-reviewed journals; (d) competitive athletic participants; and (e) a valid full- or short-form POMS questionnaire; (f) the POMS assessed before sport performance; and (g) a measure of sport performance. There was no language of publication restriction. To align with our review objectives, we gave much consideration to study participants and performance outcomes. We based the competitive athletic participants criteria on the specifics found in the sample descriptions. We did not consider participants described as recreational or intramural or volunteers at a rehabilitation clinic or exercisers. Specifically, we defined sport performance as immediate outcomes such as making an Olympic team, winning and losing sport performance statistics such as baseball batting average, and future athletic success such as becoming a professional athlete. We excluded performance outcomes associated with athletics such as shuttle run performance or vertical jump tests as they in and of themselves are not the outcome of a sporting event. Articles included in the meta-analysis portion of this review met the above inclusion requirements and provided necessary data for effect size computation. The first and third authors rigorously checked eligibility and final inclusion assessments.

### 2.3. Information Sources

We systematically identified studies by searching electronic databases, references from published POMS bibliographies, and references from two published meta-analyses. All authors conducted their electronic database search in EBSCO with the following individual databases selected: SPORTDiscus, PsycINFO, and ERIC. The main extensive search concluded in July 2019. The second author then extensively examined the search and expanded the search into January 2020.

### 2.4. Search Protocol

All authors used the following search terms: profile of mood states or POMS or iceberg profile or mental health model with sport and performance or sports performance. In EBSCO, we used the advanced search option that provided three separate boxes for search terms such as box 1 (profile of mood states), box 2 (sport), and box 3 (performance). At each search stage, we restricted EBSCO to a one-year period (e.g., 1974). Once a one-year period, each author restarted with the next year (e.g., 1975). Here are the details of our search strategy:

1. Profile of mood states, sport, performance;
2. POMS, sport, performance;
3. Iceberg profile, sport, performance;
4. Mental health model, sport, performance;
5. Profile of mood states, sport, sports performance;
6. POMS, sport, sports performance;
7. Iceberg profile, sport, sports performance;
8. Mental health model, sport, sports performance.

### 2.5. Study Selection

**Figure 1:**
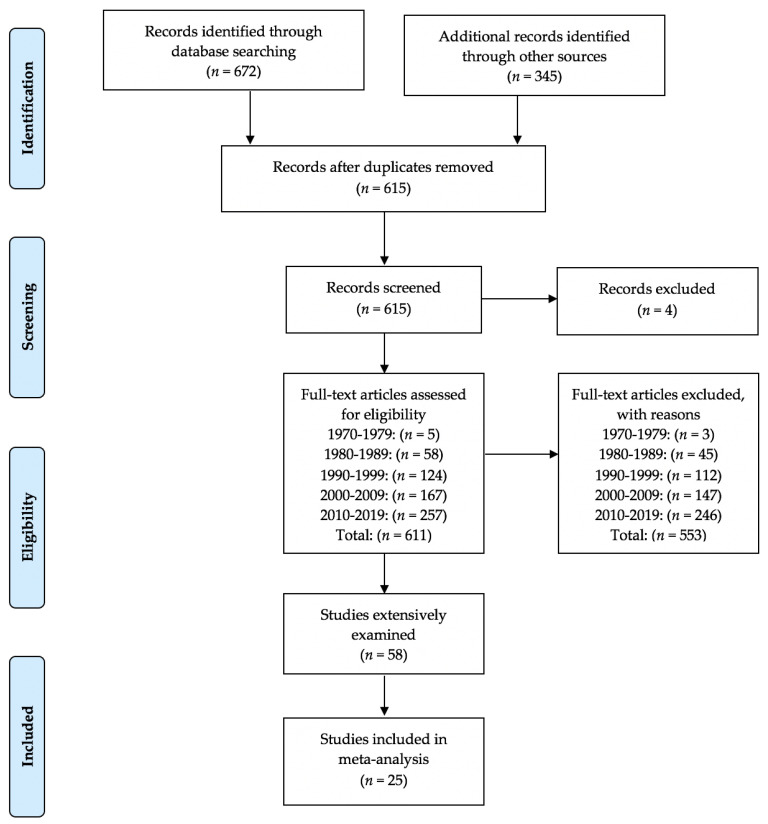
PRISMA flow chart for the identification of the eventual included studies.

As detailed in the PRISMA flow chart [28] (Figure 1) and the details of inclusion criteria, the study selection process was rigorous. Three of the authors (first, third, and fourth) engaged independently in the majority of the study selection process. The first and third author selected studies for possible inclusion while the second author engaged in a complete review of all students pulled. Through the process, we settled disagreements by consensus while examining the study inclusion criteria.

### 2.6. Data Collection Process

The first and second authors rigorously planned and carried out the data extraction process both independently and jointly. Much discussion occurred for discrepancies. All data extraction forms are available from the first author. No data or clarifications were sought from authors.

### 2.7. Data Items

To address our objectives and best understand the studies, we extracted from each study the following information: (1) sport type (including sport name, event duration, team or individual, and skill, open or closed); (2) sample characteristics (including number, percent sample male, and country); (3) study characteristics (design and confidentiality); and (4) measure characteristics (POMS wording and relation to performance, objective or self-referenced performance). For all information sought, we coded missing information as not stated. As to be detailed, some extracted information also served as study quality moderators (between study risk biases across study) as the interplay between risks of bias and studying coding influenced each other.

### 2.8. Risk of Bias in Individual Studies

The first and second author rigorously coded for the potential of individual study risk of bias. Given that studies with the POMS in sport have historically been more convenience samples than randomized and actual sport outcomes are not the result of randomized clinical trials, we examined a number of risk bias examples found in the literature [29]. After much discussion and iterations, we coded all studies on the following risks of bias: (a) sample is a close representation of the target population; (b) random selection used; (c) likelihood of non-response bias minimal; (d) performance measure relevant to the sample’s sport; (e) POMS data collected directly from the subjects; (f) reliability values reported for the POMS; (g) performance data verifiable; (h) same mode of data collection used for all subjects; (i) length of measurement time within a reasonable period between the POMS and sport performance.

### 2.9. Summary Measures

Given that both means and correlations were expected as reported data, we needed to choose a primary effect size parameter. The primary effect size measure of the relation of the POMS subscale and total mood disturbance score with sport performance was Hedges’ g [30,31]. For our overall test of the POMS and performance relationship, we used a random-effects model. For our moderator tests, we reported Hedges’ g values found in the mixed-effects analysis. Along with Hedges’ g, 95% confidence intervals, variance, and Z-values with associated *p*-values were calculated by using the Comprehensive Meta-Analysis (CMA) version-3 software (version 3.3.070, Biostat, Inc., Englewood, NJ, USA, 20 November 2014). Cohen’s (1988) interpretation for computed effect size differences criteria was used with 0.20 as small, 0.50 as medium, 0.80 as large, and 1.30 as very large.

### 2.10. Planned Methods of Analysis

Our use of random- and mixed-effects analyses meant we assumed moderate to high heterogeneity. We measured this as inconsistency (I^2^). The I^2^ statistic is the ratio of excess dispersion to total dispersion. As explained by Higgins and colleagues [32], I^2^ may be interpreted as the overlap of confidence intervals explaining the total variance attributed to the covariates. Higgins and Thompson [33] have provided a tentative classification of I^2^ values to help interpret the magnitude of the heterogeneity of variance: 25 (low), 50 (medium), and 75 (high).

We also planned for calculating standard deviations (SD) for entry into the CMA program. We calculated SD from reported means, sample sizes, and t-values in a few instances.

### 2.11. Risk of Bias across Studies

Concerning the possibility of risk of bias across studies, we examined publication bias, selective POMS scale reporting, assurance of participant anonymity, performance measure reference (objective or self), and study design concerning POMS measurement timing (more immediate or long term) on our cumulative results.

For publication bias, we examined the fail-safe *n* calculation, the funnel plot, and the ‘trim and fill’ results as calculated in the CMA program for random effects. The fail-safe *n* statistic is interpreted as the number of samples required to change a significant effect size into a non-significant effect size [34]. The greater the value, the more confidence one has that the meta-analyzed result is indeed safe from publication bias. The number of studies per reported study value was used based on the one-tail test. Thus, the larger number of studies per reported study value, the greater the confidence in the effect size being free of publication bias. Random-effects funnel plots of precision were examined to determine whether the entered studies were dispersed equally on either side of the overall effect [35] as symmetry theoretically represents the entered studies captured the essence of all relevant studies. Concerning sample size and the funnel plot, smaller studies are found closer to the bottom and larger studies closer to the top of the graph. To fix any asymmetry, Duval and Tweedie’s [36] trim and fill analysis was used. Both the number of samples needed and the resultant meta-analyzed effect size are provided in the CMA output. The first author examined each random-effects funnel plot of precision and conducted the correction analysis. The data points were either filled to the left (i.e., lowering the effect size value) or right (i.e., increasing the effect size value) of the mean, depending upon where the symmetry was lacking.

To test the impact of selective reporting (all scales, not all scales), assured participant anonymity (assured, not mentioned), performance measure (objective or self-referenced), and study design regarding the POMS measurement and performance (short- or long-term) on the cumulative results for each of the POMS scales as well as total mood disturbance, we used the CMA mixed-effects analysis. We reported the number of cases, sample sizes, and Hedges’ g, 95% confidence intervals for each level for each risk bias variable. The Q between statistic and associated *p*-value was examined to determine the statistical difference between the two levels of each risk bias moderator.

## 3. Results

### 3.1. Study Selection

From the extensive search, a total of 25 studies [16,17,18,19,20,21,37,38,39,40,41,42,43,44,45,46,47,48,49,50,51,52,53,54,55,56] met all inclusion criteria. The database search initially generated 672 citations while examining other records such as POMS bibliographies, meta-analyses, and individual studies resulted in 345 citations. After duplication removal, 615 citations remained for screening. Four articles were removed as full text or the abstracts in full were not available. Thus, we screened 611 articles for inclusion. The full text of 58 articles were screened, assessed, and debated as to whether each met all inclusion criteria. Figure 1, our flow diagram, details our complete process and indicates articles screened and removed by decade. Articles (4 from the period 1980–1989, 5 from the period 1990–1999, 12 from the period 2000–2009, and 8 from the period 2010–2019) meeting our inclusion criteria without sufficient data to analyze are available from the lead author.

### 3.2. Study Characteristics

Table 1 includes the 25 studies meeting all inclusion criteria of which four provided data for two distinctly unique samples. Overall, the 25 studies provided 32 samples with one study [40] providing samples that might have sample overlap with the reported performance measures. Hence, this study is listed only once in Table 1. From the data found in Table 1, the studies spanned from 1975 to 2011, with 1497 participants with data coming from Australia (*n* = 3), Brazil (*n* = 2), China (*n* = 1), Poland (*n* = 1), Spain (*n* = 2), Sweden (*n* = 3), the United Kingdom (*n* = 2), and the United States (*n* = 15). The majority of study participants (*n* = 18) were 100% male with only a few samples (*n* = 3) female only. Only three samples contained participants with mean ages reported as less than 18 years. Regarding the coded study characteristics, most reported mean level data (*n* = 23) and the remaining were (*n* = 6) correlational. As coded, the studies spanned performance group (*n* = 10), team selection (*n* = 9), performance outcome (*n* = 8), and future success (*n* = 2) designs. Fewer studies specifically reported anonymity assured (*n* = 13) than not reported (*n* = 16). Concerning the sport characteristics reported in Table 1, there were more individual (*n* = 17) than team sports (*n* = 12), more open- (*n* = 16) than closed- (*n* = 11)-skilled sports, and sports nearly equal in typical duration (*n* = 14 for >10 min; *n* = 15 for >10 min). The actual sports were great in variety including American football, soccer, baseball, basketball, volleyball, tennis, track, swimming, table tennis, speed skating, weightlifting, fighting, distance running, wrestling, cross-country skiing, ski marksmen, clay shooting, pentathlon, judo, and karate. Concerning coding of our measures, using mainly the POMS, the majority used the long form (*n* = 25), collected the POMS close to the actual sporting event (*n* = 22), and reported all the subscales (*n* = 20). Last, concerning the performance measure in relation to the participants, the majority were objective (*n* = 21) rather than self-referenced (*n* = 8).

### 3.3. Risk of Bias within Studies

Table 2 provides the risk of bias within studies information. The one concern would be the consistent lack of POMS reliability reporting and any form of random selection procedures. However, the researchers based their work within athletic groups and assessed participants who represented highly valued target populations (e.g., Olympic athletes, DI athletes) in very particular sports which would make random selection difficult. Thus, overall, it would seem the studies are of medium quality within our very specific inclusion criteria.

### 3.4. Results of Individual Studies, Synthesis of Results, and Risk of Bias across Studies

**Figure 2:**
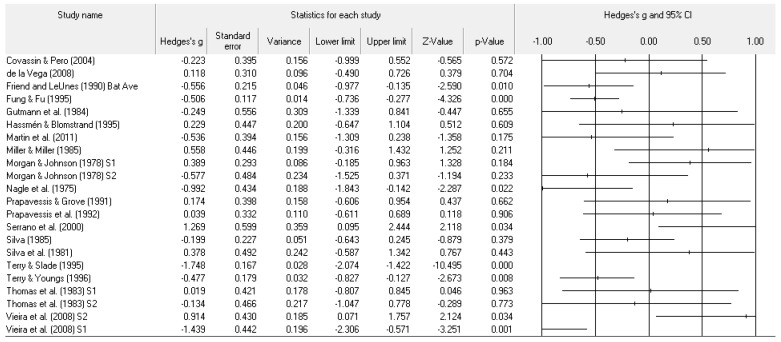
Study effect size statistics and corresponding forest plot representing the POMS tension scale, with a negative sign representing less tension for more successful performance outcomes.

**Figure 3:**
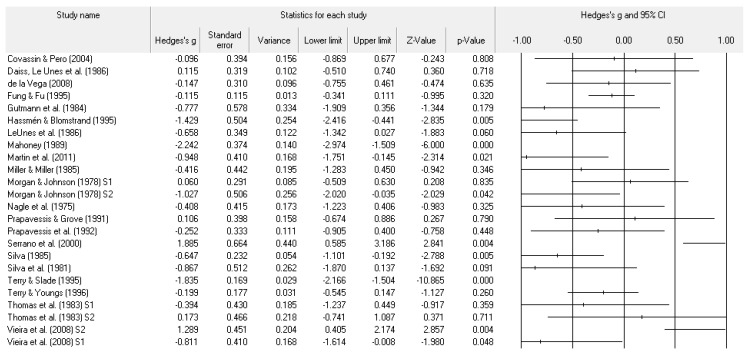
Study effect size statistics and corresponding forest plot representing the POMS depression scale, with a negative sign representing less depression for more successful performance outcomes.

**Figure 4:**
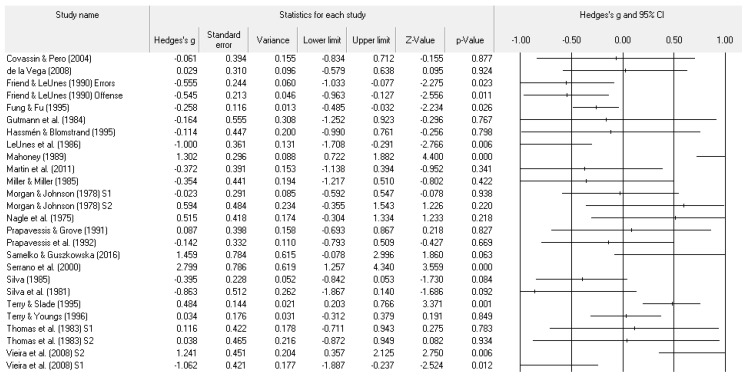
Study effect size statistics and corresponding forest plot representing the POMS anger scale, with a negative sign representing less anger for more successful performance outcomes.

**Figure 5:**
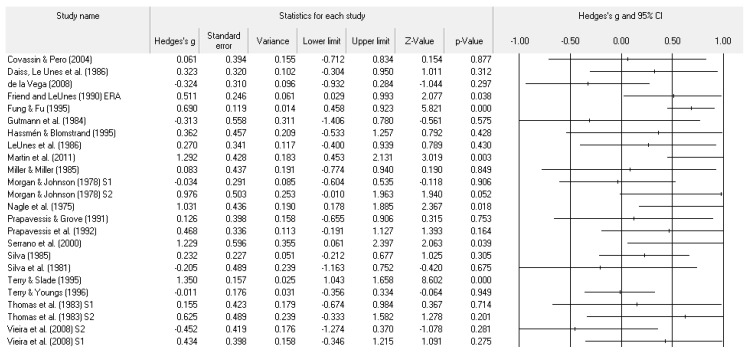
Study effect size statistics and corresponding forest plot representing the POMS vigor scale with a positive sign representing more vigor for more successful performance outcomes.

**Figure 6:**
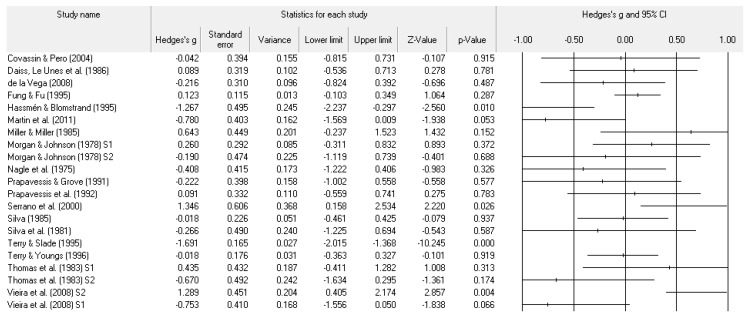
Study effect size statistics and corresponding forest plot representing the POMS fatigue scale, with a negative sign representing less fatigue for more successful performance outcomes.

**Figure 7:**
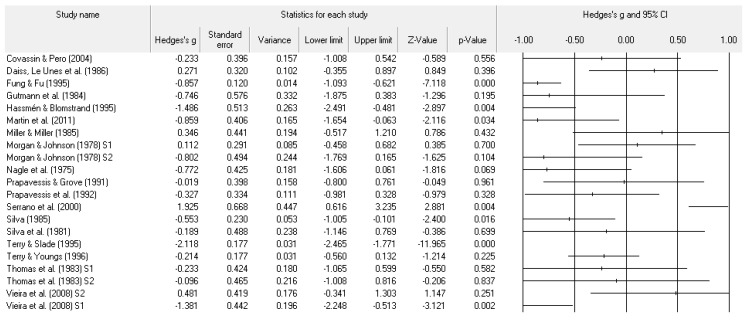
Study effect size statistics and corresponding forest plot representing the POMS confusion scale, with a negative sign representing less confusion for more successful performance outcomes.

**Figure 8:**
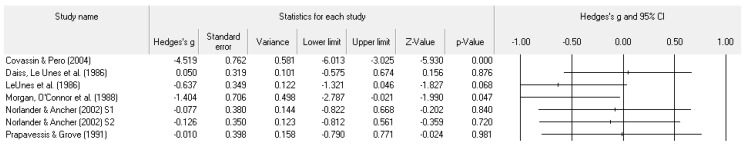
Study effect size statistics and corresponding forest plot representing the POMS TMD, with a negative sign representing less TMD for more successful performance outcomes.

A number of tables and figures at both the individual level and across all studies summarize our results. Figure 2, Figure 3, Figure 4, Figure 5, Figure 6, Figure 7 and Figure 8 contain all individual study effect size information as well as forest plots. As found in Table 3, the effect size values for depression, vigor and confusion were significantly different from zero. The effect size values were generally small. However, the upper end of each confidence interval −0.75 (depression), −0.76 (confusion), and 0.60 (vigor) was medium in meaningfulness. All effect size values except vigor had high levels of heterogeneity, verifying our upfront coding of potential moderator variables. As found in Table 4, examination of the effect size values suggests potential for some moderation, especially for sport duration. However, few statistically significant differences emerged across all the POMS scales and TMD for any of the coded moderators.

**Figure 9:**
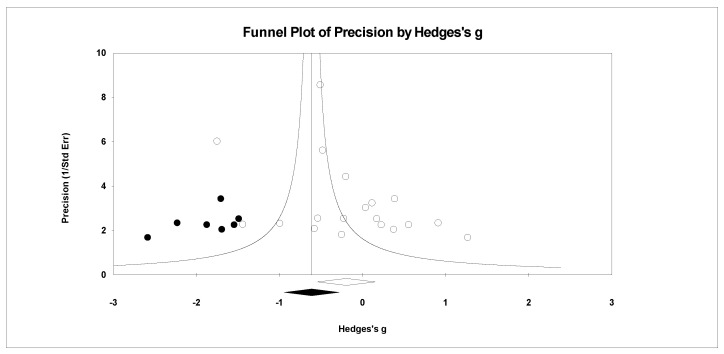
Random effects plot trimmed and filled for tension.

**Figure 10:**
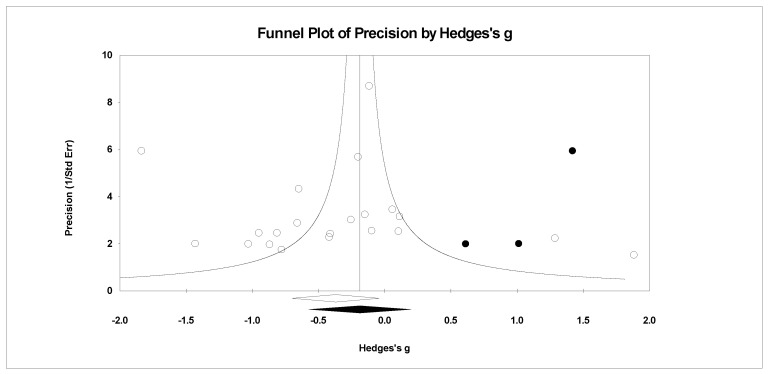
Random effects plot trimmed and filled for depression.

**Figure 11:**
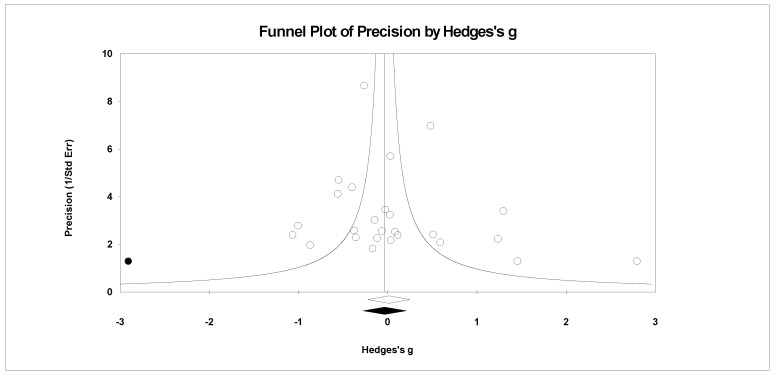
Random effects plot trimmed and filled for anger.

**Figure 12:**
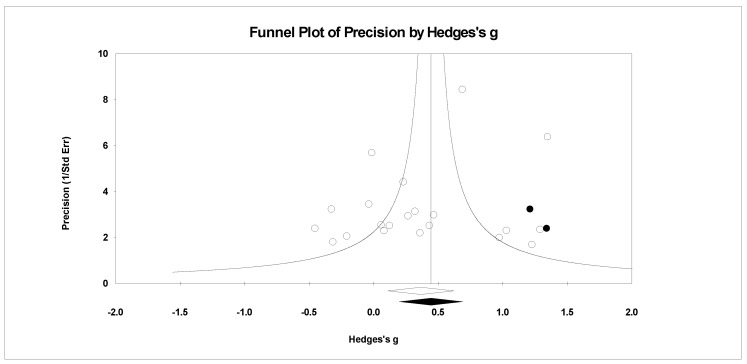
Random effects plot trimmed and filled for vigor.

**Figure 13:**
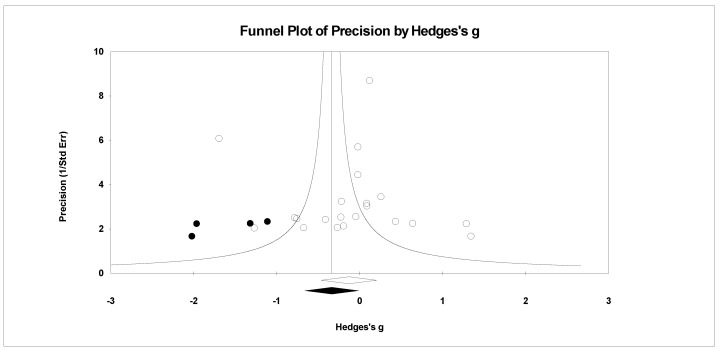
Random effects plot trimmed and filled for fatigue.

**Figure 14:**
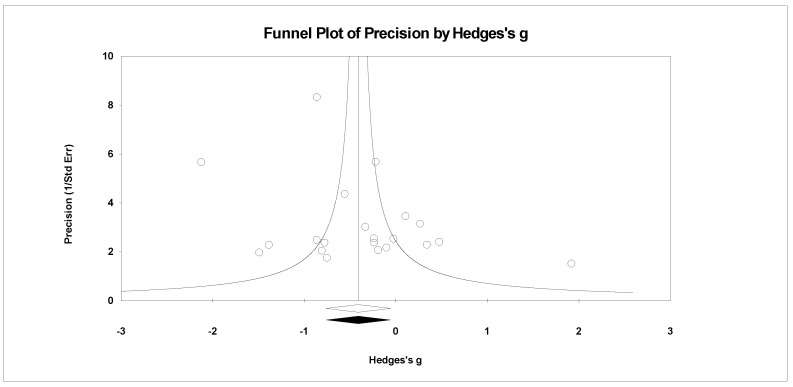
Random effects plot trimmed and filled for confusion.

**Figure 15:**
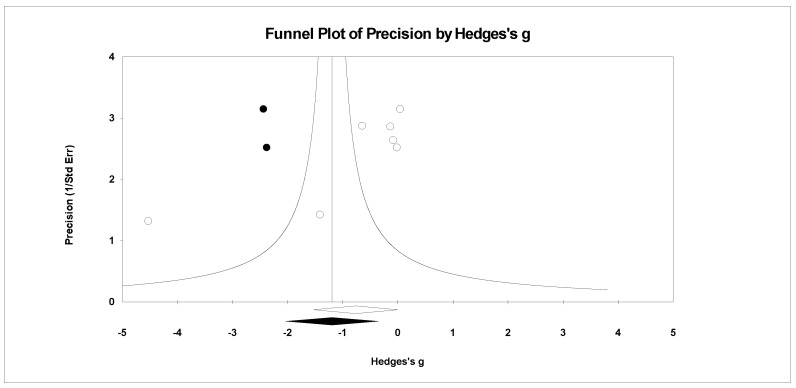
Random effects plot trimmed and filled for TMD.

Regarding the number of risk bias across study assessments, Table 4 contains the publication bias statistics and Figure 9, Figure 10, Figure 11, Figure 12, Figure 13, Figure 14 and Figure 15 are the corresponding precision plots. The fail-safe *n* values relative to the number of samples suggested that the values were free of the bias of non-significant results being “filed away” in a researcher’s office [34]. Actually, when adjusted for publication bias, TMD and all scale effect size values except for anger based on the 95% confidence intervals were reliably different from zero. Moreover, the adjusted effect size values were larger in magnitude, with TMD reaching large and depression medium in interpretation. In addition to publication bias across studies (see Table 5), we assessed four other potential sources (assured anonymity, selective POMS scale reporting, performance outcome reference, and relation of POMS measurement to performance). Though effect size values differed in some cases, no statistically significant (*p* < 0.05) differences resulted across all potential risk of bias across study analyses.

## 4. Discussion

The objective of this meta-analysis was to summarize the state of the POMS and sport performance literature to test whether Morgan’s [7,8] mental health model (i.e., higher levels of vigor and lower levels of tension, depression, anger, fatigue, and confusion) characterizes successful athletic performances and remain evens when examined across a number of potential sources of across study risk biases. In addition to our main objective, we examined Terry’s [12] propositions concerning some aspects of the sport itself would lend to greater support of Morgan’s iceberg profile. Because of our inclusion criteria, the passing of time (i.e., articles published after the past two meta-analyses), and potentially improved technology for article searching, the overlap of studies was only 7 with the Rowly [9] meta-analysis and 10 with the Beedie et al. [10] meta-analysis (included in their sport outcome results). Our results initially supported the two past meta-analyses in that overall the effect size values were general small though our TMD effect size was medium in magnitude though not reliably different from zero. Furthermore, when examining each POMS scale, our mean effect size values, except for anger, were in line with those reported by Beedie and colleagues [10] for performance outcomes. After corrected for publication bias, our mean effect size values were reliably different from zero for all but the anger scale. Of note was that the TMD effect size was large and depression medium in meaningfulness with the publication bias correction. Though certainly overall the effect sizes lend to appearances of differences, across all four of the risk bias across study moderators, none differed at the conventional *p*-value, with most being far from the conventional *p* < 0.05 value.

Our moderator analyses, though few significant results emerged, require further discussion. Terry [12] forwarded a number of propositions concerning the ability of the POMS to explain athletic performance. Specifically, the POMS subscales should explain (i.e., larger in effect size values) successful athletic performance more in short-duration sports compared to longer-duration sports, closed-skill sports compared to open-skill sports, and individual sports compared to team sports. Beedie and colleagues [10], with a colleague being Terry, tested Terry’s [12] propositions. Given their low sample sizes per moderator level, they did not test effect size statistical differences. Regardless, across each moderator and level, all effect size values were small in magnitude, ranging from 0.27 to 0.39, with the POMS scales averaged. Though still with limited samples but more than Beedie and colleagues [10], we used mixed-effects analysis to test for effect size value differences.

For duration, the effect size values themselves were in the predicted direction for tension, depression, vigor, and confusion. For anger, the *p*-value was statistically significant at the conventional level though in the opposite expected effect size direction. As discussed in the Beedie et al. [10] meta-analysis, anger could be beneficial for short-duration sports such as judo, karate, and weight lifting. When examining the individual effect size values and sports, there is support for this notion as the effect sizes for judo [49], karate [52], rowing (sample 2) [17], weight lifting [43], and wrestling [16] are all positive and ranged from 0.48 to 2.79. Further, concerning duration, though *p*-values go both ways (i.e., towards the hoped conventional significance and away from the hoped conventional significance), the effect size values for vigor seemed in line with Terry’s sport duration proposition. Therefore, the longer the sport, the more mood fluctuates and thus mood measured before performance has lower predictive power.

### 4.1. Limitations

Even though our meta-analysis process was guided by the PRISMA statement [27], a few limitations exist. First, we identified 58 studies meeting our inclusion criteria, with the POMS collected before sport performance in competitive athletes, and of those we included 25 in the analyses. As mentioned in our methodology, given the decades covered, from the 1970s to the 2020s, in our search, authors were not contacted for missing data with the foremost reason being passage of time (i.e., deceased researchers and data storage). Certainly, the across study risk bias analyses, especially the publication bias analysis, eased concern over this limitation as the publication bias analysis suggested underreporting of favorable results. Second, the number of included studies contributed to small sample sizes in our moderator analyses and limited power to detect significant between level differences as well as perhaps contributing to at time wide 95% confidence intervals. Many sports were represented but all were dependent upon POMS data reported, thus resulting in small samples for our three Terry’s proposition and risk of bias across study analyses. A better, though most likely unrealistic, research line with the POMS would be the study of one sport many times over with all POMS data reported. Third, the high amount of heterogeneity present in the data seemed to remain unexplained. Even with testing Terry’s propositions and risk of bias across study as moderators, few statistically significant results emerged. Thus, teasing out reasons for the generally high heterogeneity in the main results did not occur. We encourage Lane and Terry’s [56] conceptual model as a way to tease out high heterogeneity. Their conceptual model places depression as the most important POMS mood dimension. By splitting data on depression, Lane and Terry proposed differing answers for the other five POMS subscales and impact on subsequent performance. Fourth, given that most studies failed to report the TMD score, our reporting of data per the six scales could have posed a threat to statistical result validity as the assumption of data point independence was violated. Rowley and his colleagues [9] attempted to combat this threat by combining all of their effect size data per study to one overall effect size (they also performed the analysis again by choosing the most beneficial effect size supporting the mental health model scale score). Beedie and colleagues [10] also provided an overall mean for all six scales by computing all scales as beneficial, in that vigor remains as is and then the depression scale sign is changed as less depression, confusion and so on are characteristic of better performance. Even with this method, selective reporting of the six scales existed for the studies with summarized data in the two past meta-analyses; thus, the overall effect size for each study was not always the mean of the six scales. The best method of course is reporting the TMD mean score, standard deviation and sample size in each study.

### 4.2. Conclusions

Even with the mentioned limitations, this meta-analysis provided a better understanding of the POMS and sport performance relationships beyond that of Rowly and colleagues [9] and Beedie and colleagues [10]. We were more specific in our inclusion criteria, added many studies beyond that of the past two meta-analyses, examined a number of across studies bias risks, and used mixed-effect analyses in an attempt to examine differences in a number of potential moderators. Based on our search and review of hundreds of articles in the last decade alone, it appears that research with the POMS continued even with sometimes lackluster support [2,11,14]. It is beyond this meta-analysis to conclude whether past research suggestions have been addressed. Certainly, there is a large volume of published studies that most likely address past suggestions such as training load, within subject designs, and more youth samples. It was clear even before adjusting for potential publication bias that depression, confusion and vigor reliably impact sport performance when measured prospectively. With publication bias corrected, the POMS prediction of performance improved in magnitude and number of scale was reliably different from zero. Thus, future research with the POMS in this domain should report all data and be clear in methodology (e.g., anonymity assured). Thus, overall, especially with publication bias corrected, the POMS predicts athletic performance across a wide variety of sports and athletic performances.

## Figures and Tables

**Table 1:** Coded sample, study, sport, and measure information for all studies meeting inclusion criteria.

											Measures			
	Sample				Study		Sport				POMS			Perform
Study	*n*	Age	% M	Country	Design	Anonymity	Name	Duration	Skill	Type	Nature	Scales	Form	Relation
Covassin and Pero [37]	24	20.4	100	USA	PGBM	NR	TEN	>10	O	IND	IMMED	All, TMD	F	OBJ
Daiss et al. [38]	60	>18	100	USA	FSBM	NR	FB	>10	O	T	LT	D, V, F, C, TMD	F	OBJ
de la Vega et al. [39]	21	>18	100	ESP	PGWM	NR	SOC	>10	O	T	IMMED	T, D, A, V, F	S	OBJ
Friend and LeUnes [40]	169	20.6	100	USA	POWr	NR	BsB	>10	O	T	LT	T, A, V	F	SR
Fung and Fu [41]	300	NR	50.66	CHN	PGBM	NR	T, SWIM, TT	<10	C	IND	IMMED	All	F	OBJ
Gutmann et al. [19]	11	20.1	100	USA	TSBM	NR	SpS	<10	C	IND	IMMED	T, D, A, V, C	F	OBJ
Hassmén and Blomstrand [42]	9	22.0	100	SWE	PGWM	Yes	SOC	>10	O	T	IMMED	All	F	OBJ
LeUnes et al. [20]	33	>18	100	USA	FSBM	NR	FB	>10	O	T	LT	All, TMD	F	OBJ
Mahoney [43]	67	R	71.64	USA	POWr	NR	WL	<10	C	IND	LT	A, D	F	SR
Martin et al. [44]	25	25.8	0	USA	TSBM	NR	BkB	>10	O	T	IMMED	All	F	OBJ
Miller and Miller [22]	20	NR	0	AUS	TSBM	Yes	NB	>10	O	T	IMMED	All	F	OBJ
Morgan and Johnson [17]	57	NR	100	USA	TSBM	Yes	ROW	<10	C	T	IMMED	All	F	OBJ
	16	NR	100	USA	TSBM	Yes	ROW	<10	C	T	IMMED	All	F	OBJ
Morgan et al. [18]	14	26.4	100	USA	POWr	Yes	DR	>10	C	IND	IMMED	TMD	F	SR
Nagle et al. [16]	26	24.3	100	USA	TSBM	NR	W	<10	O	IND	IMMED	All	NR	OBJ
Norlander and Archer [45]	31	17.9	67.44	SWE	PGBM	Yes	CCS, SM	<10	B	IND	IMMED	TMD	F	OBJ
	26	17.2	50.00	SWE	PGBM	Yes	SWIM	<10	B	IND	IMMED	TMD	F	OBJ
Prapavessis and Grove [46]	12	35.6	91.66	AUS	PGWM	Yes	CS	>10	C	IND	IMMED	All, TMD	S	SR
Prapavessis et al. [47]	35	14.6	41.66	AUS	PGBM	Yes	SWIM	<10	C	IND	IMMED	All	S	SR
Samelko and Guszkowska [48]	12	20.9	58.33	POL	POWr	NR	PEN	>10	C	IND	LT	A	F	SR
Serrano et al. [49]	12	20.2	100	ESP	PGBM	NR	J	<10	O	IND	IMMED	All	F	OBJ
Silva et al. [50]	78	>18	100	USA	TSBM	NR	W	<10	O	IND	IMMED	All	F	OBJ
Silva et al. [51]	15	>18	100	USA	TSBM	NR	W	<10	O	IND	IMMED	All	F	OBJ
Terry and Slade [52]	199	26.5	100	UK	PGBM	Yes	K	<10	O	IND	IMMED	All	F	OBJ
Terry and Youngs [53]	128	20.4	50	UK	TSBM	Yes	FH	>10	O	T	IMMED	All	F	OBJ
Thomas et al. [54]	24	>18	100	USA	POWr	NR	DR	>10	C	IND	LT	All	F	SR
	20	>18	100	USA	POWr	NR	SP, JP	<10	C	IND	LT	All	F	SR
Vieira et al. [55]	12	>18	100	BRA	POWM	Yes	VB	>10	O	T	IMMED	All	F	OBJ
	12	>18	0.00	BRA	POWM	Yes	VB	>10	O	T	IMMED	All	F	OBJ

Note: S1 = sample 1 from study; S2 = sample 2 from study; R = provided age range cross both <18 and >18; NR = not reported; USA = United States of America; ESP = Spain; CHN = China; SWE = Sweden; AUS = Australia; POL = Poland; UK = United Kingdom; BRA = Brazil; PGBM = performance groups between groups mean data; FSBM = future success between groups mean data; PGWM = performance groups within groups mean data; POWr = performance outcome within groups correlational data; TSBM = team selection between groups mean data; TEN = tennis; FB = American football; SOC = soccer; BsB = baseball; T = track; SWIM = swimming; TT = table tennis; SK = speed skating; WL = weightlifting; BkB = basketball; FIGHT = fighting; NB = netball; ROW = rowing; DR = distance running; W = wrestling; CCS = cross-country skiing; SM = ski marksmen; CS = clay shooting; PEN = pentathlon; J = judo; K = karate; Sprint = track sprinters; jump = track jumpers; VB = volleyball; >10 = sport takes greater than 10 min to play; <10 = sport takes less than 10 min to play; O = open; C = closed; B = both open and closed; IND = individual; T = team; IMMED = immediate; LT = long term; A = anger; C = confusion; D = depression; F = fatigue; T = tension; V = vigor; TMD = total mood disturbance; TGM = total global mood; F = full form; S = short form; OBJ = objective; SR = self-referenced.

**Table 2:** Risk bias within studies categories information for all studies meeting inclusion criteria.

Study	Sample Close Representation of the Target Population	Random Selection Used	Non-Response Bias Minimal	Performance Measure Relevant to the Sample Sport	POMS Data Collected Directly from Subjects	POMS Reliability Values Reported	Performance Data Verifiable	Same Mode of Data Collection for All	Time Period Reasonable between the POMS and Performance
Covassin and Pero [37]	Yes	Yes	Yes	Yes	Yes	Yes	Yes	Yes	Yes
Daiss et al. [38]	No	No	Yes	Yes	Yes	No	Yes	Yes	?
de la Vega et al. [39]	Yes	No	Yes	Yes	Yes	No	Yes	Yes	Yes
Friend and LeUnes [40]	Yes	No	Yes	Yes	Yes	No	Yes	Yes	Yes
Fung and Fu [41]	Yes	Yes	Yes	Yes	Yes	No	Yes	Yes	Yes
Gutmann et al. [19]	Yes	No	Yes	Yes	Yes	No	Yes	Yes	Yes
Hassmén and Blomstrand [42]	Yes	No	Yes	Yes	Yes	No	Yes	Yes	Yes
LeUnes et al. [20]	No	No	Yes	Yes	Yes	No	Yes	Yes	?
Mahoney [43]	Yes	No	No	Yes	No	No	Yes	Yes	Yes
Martin et al. [44]	Yes	No	Yes	Yes	Yes	No	Yes	Yes	Yes
Miller and Miller [22]	Yes	No	Yes	Yes	Yes	No	Yes	Yes	Yes
Morgan and Johnson S1 [17]	Yes	No	Yes	Yes	Yes	No	Yes	Yes	Yes
Morgan and Johnson S2 [17]	Yes	No	Yes	Yes	Yes	No	Yes	Yes	Yes
Morgan et al. [18]	Yes	No	Yes	Yes	Yes	No	Yes	Yes	No
Nagle et al. [16]	Yes	No	No	Yes	Yes	No	Yes	Yes	Yes
Norlander and Archer S1 [45]	Yes	No	Yes	Yes	Yes	Yes	Yes	Yes	Yes
Norlander and Archer S2 [45]	Yes	No	Yes	Yes	Yes	Yes	Yes	Yes	Yes
Prapavessis and Grove [46]	Yes	No	Yes	Yes	Yes	No	Yes	Yes	Yes
Prapavessis et al. [47]	Yes	No	Yes	Yes	Yes	No	Yes	Yes	Yes
Samelko and Guszkowska [48]	Yes	No	Yes	Yes	Yes	No	Yes	Yes	Yes
Serrano et al. [49]	Yes	Yes	Yes	Yes	?	No	Yes	Yes	Yes
Silva et al. [50]	Yes	No	Yes	Yes	Yes	No	Yes	Yes	Yes
Silva et al. [51]	Yes	No	Yes	Yes	Yes	No	Yes	Yes	Yes
Terry and Slade [52]	Yes	No	?	Yes	Yes	No	Yes	Yes	Yes
Terry and Youngs [53]	Yes	No	Yes	Yes	Yes	No	No	Yes	Yes
Thomas et al. S1 [54]	Yes	No	Yes	Yes	Yes	No	Yes	Yes	Yes
Thomas et al. S2 [54]	Yes	No	Yes	Yes	Yes	No	Yes	Yes	Yes
Vieira et al. S1 [55]	Yes	Yes	Yes	Yes	Yes	No	Yes	Yes	Yes
Vieira et al. S2 [55]	Yes	Yes	Yes	Yes	Yes	No	Yes	Yes	Yes

**Table 3:** Summary effect size, heterogeneity, and publication bias statistics for all POMS scales and TMD.

	Overall Effect Size Information				Heterogeneity		Publication Bias		
Scale	k	*n*	Hedges’ g [95% CI]	Z-value [*p*]	I^2^	tau^2^	Fail-Safe *n*	Trim *n*	Adjusted g [Adjusted 95% CI]
Tension	22	1150	−0.21 [−0.51, 0.09]	−1.24 [0.216]	81.89	0.38	146	6	−0.47 [−0.76, −0.19]
Depression	24	1215	−0.43 [−0.75, −0.11]	−2.36 [0.018]	84.59	0.50	386	4	−0.64 [−0.97, −0.31]
Anger	30	1689	0.08 [−0.15, 0.30]	0.38 [0.702]	75.85	0.25	30	0	No change
Vigor	24	1220	0.38 [0.15, 0.60]	3.01 [0.001]	68.69	0.18	332	2	0.44 [0.22, 0.67]
Fatigue	21	1104	−0.13 [−0.46, 0.20]	−0.67 [0.501]	84.39	0.46	27	4	−0.34 [−0.66, −0.01]
Confusion	21	1094	−0.41 [−0.76, −0.06]	−2.11 [0.035]	85.08	0.52	365	0	No change
TMD	9	257	−0.53 [−1.14, 0.07]	−1.53 [0.125]	80.81	0.66	24	2	−0.84 [−1.49, −0.18]

**Table 4:** Terry’s propositions for sport characteristic results.

Scale	Moderator Levels	k	*n*	Hedges’ g [95% CI]	QTB [*p*]
Sport Duration					
Tension	<10, >10	10, 10	749, 357	−0.28 [−0.80, 0.24], −0.16 [−0.51, 0.19]	0.14 [0.71]
Depression	<10, >10	11, 11	816, 355	−0.62 [−1.19, −0.05], −0.27 [−0.61, 0.07]	1.05 [0.30]
Anger	<10, >10	13, 15	1030, 615	0.31 [−0.04, 0.65], −0.15 [−0.43, 0.13]	4.01 [0.04]
Vigor	<10, >10	10, 12	749, 427	0.57 [0.21, 0.93], 0.20 [−0.03, 0.42]	2.89 [0.09]
Fatigue	<10, >10	9, 10	738, 322	−0.13 [−0.73, 0.47], −0.12 [−0.48, 0.24]	0.00 [0.98]
Confusion	<10, >10	10, 9	749, 301	−0.52 [−1.07, 0.03], −0.30 [−0.70, 0.10]	0.42 [0.52]
TMD	<10, >10	4, 5	114, 143	0.02 [−0.34, 0.38], −1.14 [−2.29, 0.01]	3.58 [0.06]
Sport Skill					
Tension	Closed, Open	8, 14	475, 675	−0.13 [−0.44, 0.18], −0.25 [−0.70, 0.20]	0.18 [0.67]
Depression	Closed, Open	9, 15	542, 673	−0.47 [−0.92, −0.00], −0.39 [−0.84, 0.05]	0.05 [0.82]
Anger	Closed, Open	10, 20	554, 1135	0.23 [−0.16, 0.62], 0.01 [−0.27, 0.30]	0.77 [0.38]
Vigor	Closed, Open	8, 16	475, 745	0.40 [0.13, 0.69], 0.38 [0.06, 0.70]	0.01 [0.93]
Fatigue	Closed, Open	7, 14	464, 640	0.09 [−0.09, 0.27], −0.19 [−0.66, 0.29]	1.11 [0.29]
Confusion	Closed, Open	8, 13	475, 619	−0.39 [−0.73, −0.03], −0.41 [−0.97, 0.15]	0.01 [0.94]
TMD	Closed, Open	2, 3	26, 117	−0.58 [−1.93, 0.76], −1.55 [−3.43, 0.33]	0.67 [0.41]
Sport Type					
Tension	IND, Team	12, 10	756, 394	−0.25 [−0.70, 0.20], −0.15 [−0.52, 0.22]	0.11 [0.74]
Depression	IND, Team	13, 11	823, 392	−0.47 [−0.94, 0.03], −0.33 [−0.68, 0.02]	0.20 [0.65]
Anger	IND, Team	16, 14	1049, 640	0.29 [−0.02, 0.60], −0.17 [−0.45, 0.11]	4.47 [0.03]
Vigor	IND, Team	12, 12	756, 464	0.51 [0.18, 0.83], 0.23 [−0.01, 0.48]	1.76 [0.18]
Fatigue	IND, Team	11, 10	745, 359	−0.16 [−0.70, 0.37], −0.08 [−0.44, 0.28]	0.06 [0.80]
Confusion	IND, Team	12, 9	756, 338	−0.44 [−0.93, 0.05], −0.33 [−0.74, 0.08]	0.12 [0.72]
TMD	IND, Team	7, 2	164, 93	−0.66 [−1.49, 0.16], −0.28 [−0.95, 0.39]	0.50 [0.48]

**Table 5:** Number of samples (k), number of participants, Hedges’ g, confidence intervals, and the Q total between statistics for potential risk of bias across study variables.

Scale	Moderator Level	k	*n*	Hedges’ g [95% CI]	QTB [*p*-Value]
Anonymity Assured Written in Text					
Tension	Yes, NR	10, 12	499, 649	−0.22 [−0.84, 0.40], −0.26 [−0.50, −0.02]	0.02 [0.89]
Depression	Yes, NR	10, 14	499, 714	−0.46 [−1.61, 0.15], −0.41 [−0.77, −0.06]	0.02 [0.90]
Anger	Yes, NR	10, 20	499, 1188	0.09 [−0.23, 0.40], 0.09 [−0.21, 0.39]	0.00 [1.00]
Vigor	Yes, NR	10, 14	499, 719	0.34 [−0.11, 0.79], 0.39 [0.16, 0.63]	0.04 [0.83]
Fatigue	Yes, NR	10, 11	499, 603	−0.20 [−0.89, 0.43], −0.04 [−0.27, 0.19]	0.22 [0.64]
Confusion	Yes, NR	10, 11	499, 593	−0.54 [−1.20, 0.12], −0.32 [−0.68, 0.04]	0.33 [0.56]
TMD	Yes, NR	6, 3	140, 117	−0.08 [−0.45, 0.29], −1.55 [−3.43, 0.33]	2.25 [0.13]
Selective Reporting of POMS Scales					
Tension	Yes, No	3, 19	127, 1021	−0.28 [−0.74, 0.19], −0.20 [−0.54, 0.14]	0.07 [0.79]
Depression	Yes, No	4, 20	159, 1054	−0.75 [−1.84, 0.34], −0.37 [−0.71, −0.03]	0.42 [0.51]
Anger	Yes, No	10, 20	633, 1054	0.28 [−0.18, 0.74], −0.02 [−0.26, 0.22]	1.26 [0.26]
Vigor	Yes, No	4, 20	164, 1054	0.13 [−0.30, 0.56], 0.43 [0.18, 0.68]	1.34 [0.24]
Fatigue	Yes, No	2, 19	81, 1021	−0.07 [−0.50, 0.36], −0.14 [−0.50, 0.23]	0.06 [0.80]
Confusion	Yes, No	2, 19	71, 1021	−0.13 [−1.10, 0.84], −0.43 [−0.80, −0.07]	0.34 [0.56]
TMD	Yes, No	6, 3	188, 69	−0.06 [−0.42, 0.30], −1.59 [−3.56, 0.37]	2.25 [0.13]
Sport Performance Relation to Participants					
Tension	Objective, Self	17, 5	962, 186	−0.23 [−0.60, 0.14], −0.21 [−0.51, 0.09]	0.01 [0.94]
Depression	Objective, Self	19, 5	1055, 158	−0.40 [−0.76, −0.04], −0.53 [−1.43, 0.36]	0.07 [0.78]
Anger	Objective, Self	20, 10	1209, 478	0.06 [−0.20, 0.31], 0.14 [−0.30, 0.59]	0.11 [0.74]
Vigor	Objective, Self	19, 5	1055, 163	0.37 [0.09, 0.64], 0.41 [0.10, 0.71]	0.03 [0.86]
Fatigue	Objective, Self	17, 4	1011, 91	−0.14 [−0.53, 0.24], −0.04 [−0.45, 0.36]	0.12 [0.73]
Confusion	Objective, Self	17, 4	1001, 91	−0.46 [−0.89, −0.05], −0.19 [−0.58, 0.20]	0.89 [0.34]
TMD	Objective, Self	7, 2	231, 26	−0.53 [−1.27, 0.19], −0.58 [−1.93, 0.76]	0.00 [0.94]
Timing of POMS in Relation to Sport Performance					
Tension	Short, Long term	19, 3	1009, 139	−0.20 [−0.52, 0.15], −0.39 [−0.71, −0.05]	0.62 [0.43]
Depression	Short, Long term	19, 5	1009, 204	−0.38 [−0.73, −0.02], −0.61 [−1.48, 0.26]	0.22 [0.63]
Anger	Short, Long term	21, 9	1223, 464	0.10 [−0.14, 0.33], 0.07 [−0.45, 0.59]	0.01 [0.93]
Vigor	Short, Long term	19, 5	1009, 209	0.37 [0.10, 0.65], 0.39 [0.10, 0.68]	0.01 [0.93]
Fatigue	Short, Long term	18, 3	998, 104	−0.15 [−0.52, 0.22], 0.00 [−0.55, 0.55]	0.20 [0.65]
Confusion	Short, Long term	18, 3	988, 104	−0.48 [−0.86, −0.09], 0.05 [−0.39, 0.48]	3.07 [0.08]
TMD	Short, Long term	7, 2	164, 93	−0.66 [−1.49, 0.16], −0.28 [−0.95, 0.39]	0.50 [0.48]

## Data Availability

The data presented in this study are available on request from the corresponding author in either MS Excel or the Comprehensive Meta-Analysis program format.
