# Correction: Lochbaum et al. The Profile of Moods States and Athletic Performance: A Meta-Analysis of Published Studies. *Eur. J. Investig. Health Psychol. Educ.* 2021, *11*, 50–70

**DOI:** 10.3390/ejihpe11020036

**Published:** 2021-06-09

**Authors:** Marc Lochbaum, Thaís Zanatta, Deylon Kirschling, Emily May

**Affiliations:** 1 Department of Kinesiology and Sport Management, Texas Tech University, Lubbock, TX 79409, USA; 2 Education Academy, Vytautas Magnus University, 44248 Kaunas, Lithuania; 3 Psychological Sciences, University of California, Merced, CA 95343, USA; tbenoit2@ucmerced.edu; 4 College of Rehabilitative Sciences, University of St. Augustine Health Sciences, Austin, TX 78739, USA; d.kirschling@usa.edu; 5 School of Health Professions, University of Texas Medical Branch, Galveston, TX 77555, USA; ecmay@utmb.edu

Error in Section 2.6

In the original article [1], there was a mistake in Section 2.6 as published. The wrong paragraph was presented. The authors wish to remove the whole paragraph. The corrected Section 2.6 appears below. The authors apologize for any inconvenience caused and state that the scientific conclusions are unaffected. The original article has been updated.

*2.6. Data Collection Process*

The first and second authors rigorously planned and carried out the data extraction process both independently and jointly. Much discussion occurred for discrepancies. All data extraction forms are available from the first author. No data or clarifications were sought from authors.

Error in Section 3.1

In the original article [1], there were two mistakes in Section 3.1 as published. (1) The authors wish to remove the sentence “Research manuscripts reporting large datasets that are deposited in a publicly available database.” in Section 3.1. (2) The whole paragraph under Section 3 should be moved under the Section 3.1. The corrected Section 3.1 appears below. The authors apologize for any inconvenience caused and state that the scientific conclusions are unaffected. The original article has been updated.

**3. Results**

*3.1. Study Selection*

From the extensive search, a total of 25 studies [16–21,37–56] met all inclusion criteria. The database search initially generated 672 citations while examining other records such as POMS bibliographies, meta-analyses, and individual studies resulted in 345 citations. After duplication removal, 615 citations remained for screening. Four articles were removed as full text or the abstracts in full were not available. Thus, we screened 611 articles for inclusion. The full text of 58 articles were screened, assessed, and debated as to whether each met all inclusion criteria. Figure 1, our flow diagram, details our complete process and indicates articles screened and removed by decade. Articles (4 from the period 1980–1989, 5 from the period 1990–1999, 12 from the period 2000–2009, and 8 from the period 2010–2019) meeting our inclusion criteria without sufficient data to analyze are available from the lead author.

The authors apologize for any inconvenience caused and state that the scientific conclusions are unaffected. The original article has been updated.
